# Mixed effects approach to the analysis of the stepped wedge cluster randomised trial - accounting for the confounding effect of time

**DOI:** 10.1186/1745-6215-16-S2-P142

**Published:** 2015-11-16

**Authors:** Alecia Nickless, Merryn Voysey, Ly-Mee Yu

**Affiliations:** 1 Nuffield Department of Primary Care Health Sciences, University of Oxford, Oxford, UK

## 

The stepped wedge cluster randomised trial is becoming more popular, as it is both logistically more viable for large-scale intervention roll-outs than the conventional parallel cluster randomised trial, and can be more ethically responsible when it is perceived that the intervention will do more good than harm. Stepped wedge designs have an inherent time component due to the staggered nature of the design. Although it is possible to analyse the data by ignoring time, for example through the use of a paired analysis, these approaches may result in inaccurate conclusions if there is a time trend in the data. This can occur, for example, when there is a general initiative to improve service, resulting in improvement across both the control and intervention periods over time. If time is not accounted for, there may appear to be a treatment effect where none exists. We compare different mixed effect model formulations, as well as a simple paired analysis, to describe the options available when formulating the mixed effects model, to account for time trends and obtain results which appropriately depict the stepped wedge cluster randomised trial design. These different approaches are illustrated using data from the OXTEXT-7 evaluation of “Feeling Well with True Colours” initiative implemented by adult community mental health teams in the Oxford Health NHS Foundation Trust.

